# Supplementary material for: Mutations in PCYT2 disrupt etherlipid biosynthesis and cause a complex hereditary spastic paraplegia
Source: Brain. 2019 Oct 22;142(11):3382–97. doi: 10.1093/brain/awz291 (PMC6821184; doi:10.1093/brain/awz291)
Supplement: awz291_Supplementary_Data [file awz291_supplementary_data.zip › awz291-Suppl_data/Supplementary_Data4.zip!/Supplementary information 3 - fibroblast lipidomics.zip!/Fibroblast lipidomics/Multiple comparison/PLS-DA.pdf]

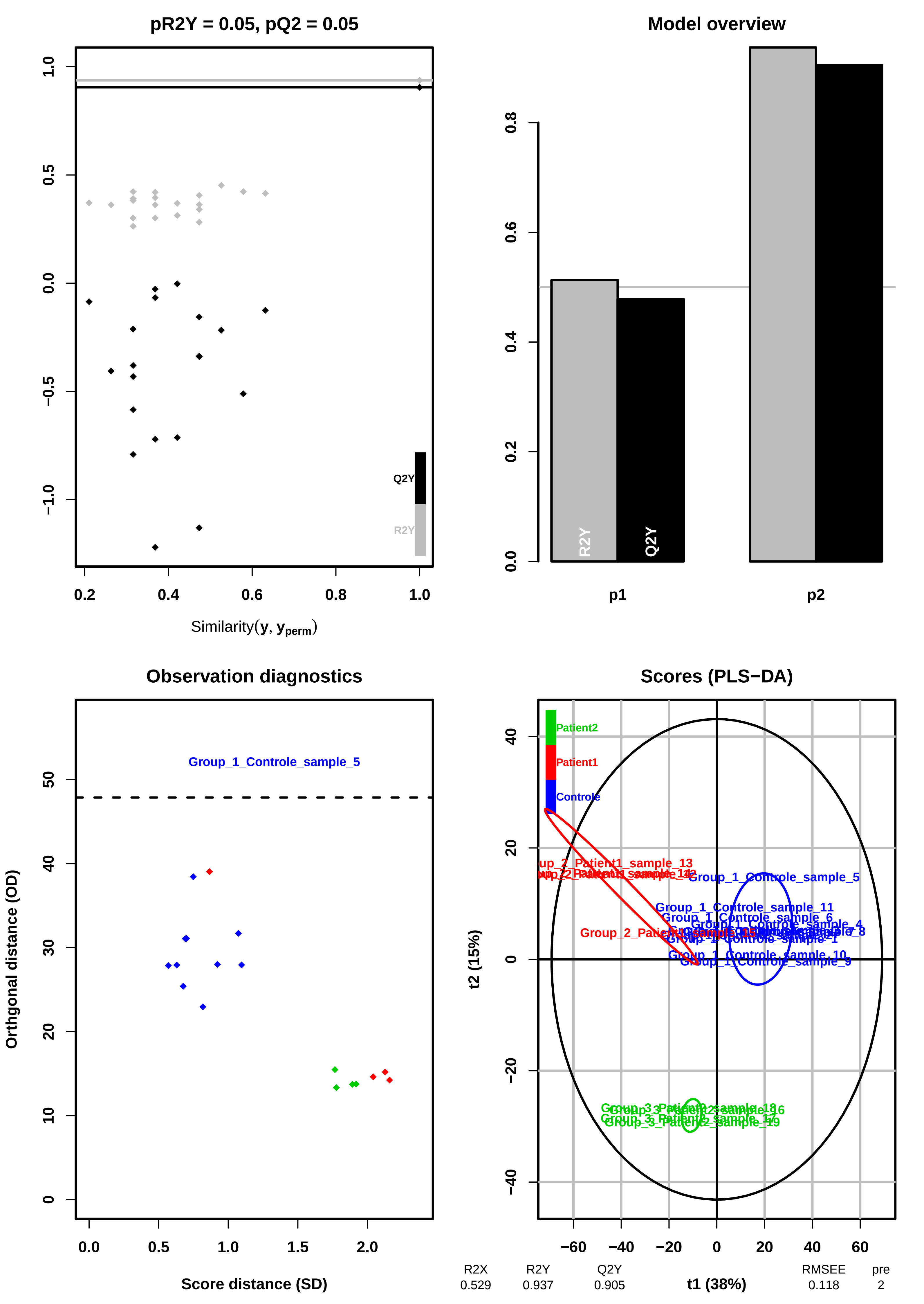
pR2Y = 0.05, pQ2 = 0.05
Q2Y
R2Y
Similarity(y, yperm)
Model overview
R2Y
Q2Y
p1
p2
Observation diagnostics
Group_1_Controle_sample_5
Orthgonal distance (OD)
Score distance (SD)
Scores (PLS−DA)
Patient2
Patient1
Controle
Group_1_Controle_sample_5
Group_1_Controle_sample_11
Group_1_Controle_sample_10
Group_1_Controle_sample_9
t2 (15%)
t1 (38%)
R2X 0.529
R2Y 0.937
Q2Y 0.905
RMSEE 0.118
pre 2
